# Calcium ions function as a booster of chromosome condensation

**DOI:** 10.1038/srep38281

**Published:** 2016-12-02

**Authors:** Rinyaporn Phengchat, Hideaki Takata, Kenichi Morii, Noriko Inada, Hideji Murakoshi, Susumu Uchiyama, Kiichi Fukui

**Affiliations:** 1 Graduate School of Engineering, Osaka University, 2-1 Yamadaoka, Suita 565-0871, Osaka, Japan; 2 The Graduate School of Biological Sciences, Nara Institute of Science and Technology, 8916-5 Takayama-Cho Ikoma-shi, Nara 630-0192, Japan; 3 Supportive Center for Brain Research, National Institute for Physiological Sciences, Okazaki, Aichi 444-8585, Japan

## Abstract

Chromosome condensation is essential for the faithful transmission of genetic information to daughter cells during cell division. The depletion of chromosome scaffold proteins does not prevent chromosome condensation despite structural defects. This suggests that other factors contribute to condensation. Here we investigated the contribution of divalent cations, particularly Ca^2+^, to chromosome condensation *in vitro* and *in vivo*. Ca^2+^ depletion caused defects in proper mitotic progression, particularly in chromosome condensation after the breakdown of the nuclear envelope. Fluorescence lifetime imaging microscopy-Förster resonance energy transfer and electron microscopy demonstrated that chromosome condensation is influenced by Ca^2+^. Chromosomes had compact globular structures when exposed to Ca^2+^ and expanded fibrous structures without Ca^2+^. Therefore, we have clearly demonstrated a role for Ca^2+^ in the compaction of chromatin fibres.

The formation of a metaphase chromosome is a key process in gene organization. Since the discovery of chromosomes in the 19^th^ century, scientists have been attempting to clarify how they transit from a decondensed structure during interphase to a condensed structure during mitosis. Chromosomes are composed of nucleosomes, which are composed of DNA strands wrapped around a histone octamer^1^,^2^. Nucleosomes are 11 nm in diameter appear as ‘beads’ in ‘beads-on-a-string’ structure in low-salt solutions^3^. However, the organization of metaphase chromosomes is still controversial. There are three major models describing the formation of metaphase chromosomes from chromatin fibres^4^: (1) a coiled coil model, which is a stepwise progression from nucleosome fibres to 30 nm chromatin fibres to thicker chromatin fibres^5^; (2) a radial loop model, where loops of chromatin fibres are formed around a chromosome scaffold^6^,^7^; and (3) a folded fibre model, where chromatin fibres folded irregularly into chromosomes^8^.

Three factors are known to be important for the formation and maintenance of chromosome structure: chromosomal scaffold proteins, histone post-translational modifications (PTMs), and cations^9^,^10^,^11^,^12^. Major scaffold proteins involved in chromosome organization include condensins, topoisomerase IIα (Topo IIα), and kinesin family 4 (KIF4)^11^,^13^. Although depletion of these proteins disrupts chromosome structure, X-shaped chromosomes are still formed^13^,^14^. This suggests that scaffold proteins are not the only factors contributing to chromosome condensation. Histone PTMs promote nucleosome–nucleosome interaction by modifying the surface charge of histones or by recruiting other proteins to bind to nucleosomes^11^,^15^,^16^. Cations may promote chromosome condensation by cancelling the negative charge of DNA^17^,^18^,^19^. The monovalent cations, divalent cations, and polyamines that promote nucleosome folding have been identified by nucleosome arrays^18^,^20^. However, unlike *in vitro* systems using reconstituted chromatins, chromatin fibres might behave differently in living cells because chromatin fibres exist as high density stages in a cell and they are surrounded by macromolecules and various electrolytes.

Divalent cations, particularly Mg^2+^ and Ca^2+^, are known to increase and shift from their storage organelles to chromatin during the mitotic phase of the cell cycle^21^,^22^, implicating them in mitosis. Unlike Mg^2+^, free intracellular Ca^2+^ is present in low concentrations (nanomolar level) and is tightly regulated. As a secondary messenger, Ca^2+^ plays an important role in activating or inhibiting many intracellular signalling cascades including those that control cell cycle^23^,^24^. However, the precise roles in mitotic chromosome condensation remain to be elucidated. Cations in cryofractured mitotic cells identified by secondary ion mass spectrometry (SIMS) revealed a higher concentration of Ca^2+^ (12–24 mM) on metaphase chromosomes compared with interphase nuclei (4–6 mM)^22^. Levels of chromatin-bound Ca^2+^ are much higher than levels of Mg^2+^ in the cytosol (2–3 mM) and on chromosomes (5–17 mM)^22^. This suggests that Ca^2+^ plays a more important role than Mg^2+^ in chromosome compaction. In this study, we investigated the role of divalent cations, particularly Ca^2+^, in chromosome condensation *in vitro* and *in vivo*. Using fluorescence lifetime imaging microscopy-Förster resonance energy transfer (FLIM-FRET), we demonstrated that chromosome compaction is influenced by changes in Ca^2+^ concentration. Detailed chromosome structures affected by changes in Ca^2+^ concentration were examined at high resolution using electron microscopy. Our findings demonstrated that Ca^2+^ is important for the organization of metaphase chromosomes.

## Results

### Effect of calcium ion depletion on mitotic progression

**Figure 1:**
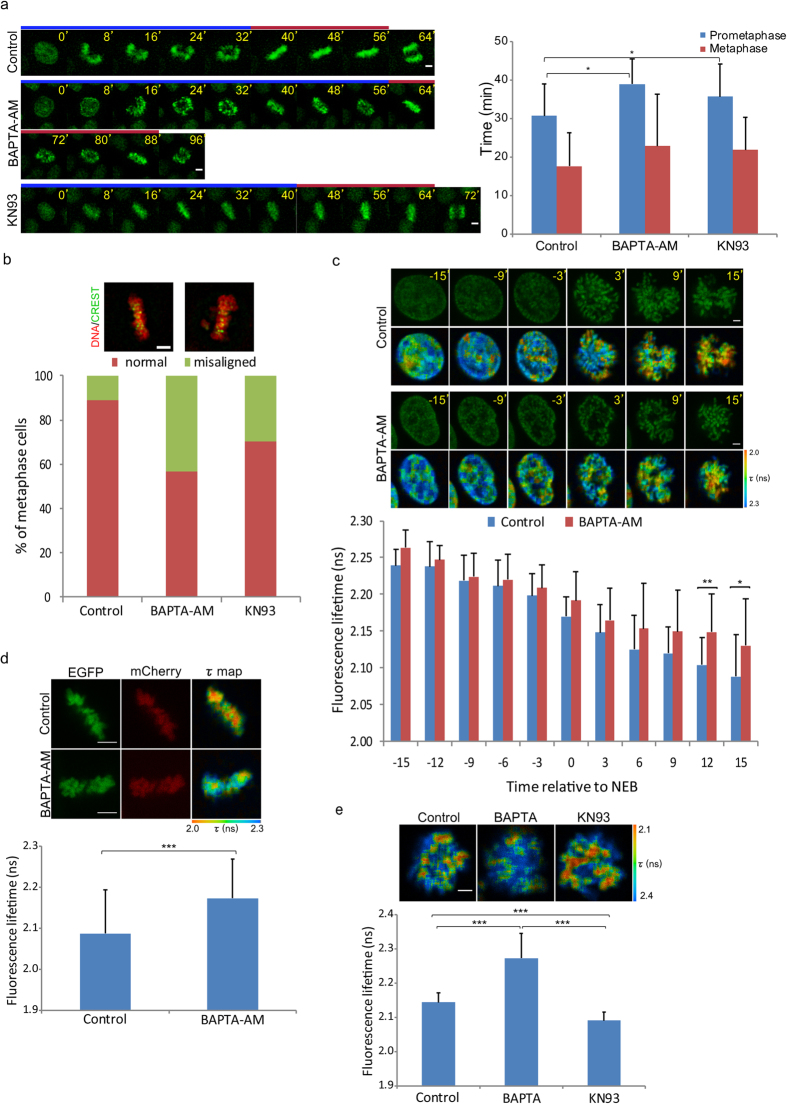
Effect of Ca^2+^ depletion on mitotic progression. (**a**) Mitotic progression in control (*n* = 15 cells), BAPTA-AM-treated (*n* = 23 cells) and KN93-treated (*n* = 32 cells) cells monitoring from nuclear envelop breakdown (NEB, set at *t* = 0 min) to the onset of anaphase. BAPTA-AM is a membrane permeable Ca^2+^-chelating agent while KN93 is an inhibitor of CaMKII. The H2B-EGFP signal was detected to visualize chromatin. (**b**) Defects in chromosome alignment in control and Ca^2+^-depleted cells (*n* > 150 cells). (**c**) Chromosome compaction during mitosis was quantified by FLIM-FRET analysis using 2P-FLIM with 880 nm excitation. Chromosome condensation was observed from prophase to prometaphase. NEB was set at *t* = 0 min. Pseudocolours represent fluorescence lifetime (τ) of EGFP. Stacked columns showed mean fluorescence lifetime from 13 cells for control and Ca^2+^-depletion. (**d**) Compaction of chromosomes in control (*n* = 27 cells) and Ca^2+^-depleted (*n* = 27 cells) metaphase cells. (**e**) Compaction of chromosomes in nocodazole-arrested cells treated with DMSO (*n* = 24 cells), BAPTA (with ionomycin, *n* = 19 cells) or KN93 (*n* = 31 cells). Error bars indicate standard deviations. Bar, 5 μm.

To examine the effect of Ca^2+^ depletion on mitotic progression, we treated HeLa^H2B-2FP^ cells co-expressing EGFP- and mCherry-tagged histone H2B with the Ca^2+^-chelating agent BAPTA-AM. Cell observation started with the onset of nuclear envelope breakdown (NEB), which was set as *t* = 0 min and continued until anaphase when sister chromatids were separated. Prometaphase and metaphase were determined based on chromosome morphology by observing EGFP and mCherry signals, specifically bound to chromatin as previously described^25^. Depletion of intracellular Ca^2+^ prolonged the prometaphase stage of mitosis (Fig. 1a). Although the average metaphase duration was slightly longer following Ca^2+^ depletion, this was not statistically significant. More than one quarter of Ca^2+^-depleted cells took more than 90 min to proceed from NEB to anaphase (Supplementary Fig. 1). In addition, depletion of Ca^2+^ during mitosis led to a threefold increase in the number of cells with chromosome misalignment (Fig. 1b). In agreement with previous reports^26^,^27^, these results clearly indicated that Ca^2+^ is required for proper mitotic progression.

Previous reports have shown that chromosome condensation occurs before and during NEB and that compaction of chromosomes dramatically increased after NEB^28^,^29^. High amount of chromatin-bound Ca^2+^ was also detected on prometaphase and metaphase chromosome spreads^30^, implicating Ca^2+^ in global chromosome compaction. Defects in chromosome condensation might induce mitotic delay^28^. Therefore, we monitored chromosome compaction during the early phases of mitosis using FLIM-FRET following depletion of intracellular Ca^2+^ by BAPTA-AM. Chromatin compaction was quantified in HeLa^H2B-2FP^ cells by FLIM-FRET using EGFP and mCherry as a FRET pair, with EGFP acting as the donor fluorophore^31^,^32^. Fluorescence lifetime of EGFP was acquired using time-correlated single photon counting (TCSPC)-FLIM. Chromosome condensation brings EGFP and mCherry close enough for energy transfer from excited EGFP to mCherry, resulting in a shorter fluorescence lifetime (τ) of EGFP. With this approach, chromosome condensation could be properly monitored in HeLa^H2B-2FP^ cells *in vivo*.

Chromosome condensation occurred before NEB in both control and Ca^2+^-depleted cells, decreasing fluorescence lifetime of EGFP (Fig. 1c). At *t* = 0 min (onset of NEB), Ca^2+^-depleted cells had longer fluorescence lifetime indicating that chromosomes were less compact compared with control cells. Chromosome condensation continued after NEB, and the dynamics were clearly distinguishable between control and Ca^2+^-depleted cells. From twelve minutes after NEB, mean fluorescence lifetime of EGFP in Ca^2+^-depleted cells was significantly longer than the control cells indicating that chromosomes were less compact.

The prolonged prometaphase stage in Ca^2+^-depleted cells might delay chromosome condensation (Fig. 1a). Therefore, we compared chromosome compaction at metaphase in control and Ca^2+^-depleted cells. Treatment with the proteasome inhibitor MG132 arrested HeLa^H2B-2FP^ cells at metaphase without disturbing spindle fibres. Interestingly, metaphase chromosomes in Ca^2+^-depleted cells were still less compact than control cells (Fig. 1d), having longer fluorescence lifetime of EGFP. As a control experiment, chromosome compaction in condensin-depleted cells was quantified by FLIM-FRET. Consistent with previous studies^13^,^14^,^28^, depletion of hCAP-E, a condensin subunit, significantly increased fluorescence lifetime of EGFP indicating chromosome decondensation (Supplementary Fig. 2). Taken together, these results suggest that Ca^2+^ plays an essential role in the condensation of chromosomes during mitosis.

Calcium/calmodulin-stimulated protein kinase II (CaMKII) is a Ca^2+^-sensing protein that regulates cell cycle progression. CaMKII activity is required for mitotic entry^33^,^34^ and metaphase-anaphase transition^24^. Depletion of Ca^2+^ might inhibit CaMKII activation, affecting its downstream effector proteins, leading to chromosome decompaction. Inhibition of CaMKII activity using KN93 prolonged the prometaphase stage and increased the number of cells with misaligned chromosomes, similar to Ca^2+^ depletion (Fig. 1a,b). However, in contrast to Ca^2+^ depletion, CaMKII inhibition reduced the fluorescence lifetime of H2B-EGFP, suggesting chromosome hypercompaction (Fig. 1e). These results indicate that chromosome decompaction is directly induced by Ca^2+^ depletion, and that CaMKII is unlikely to be involved in the process.

### Alteration of chromosome compaction *in vivo* by manipulating intracellular calcium level

**Figure 2:**
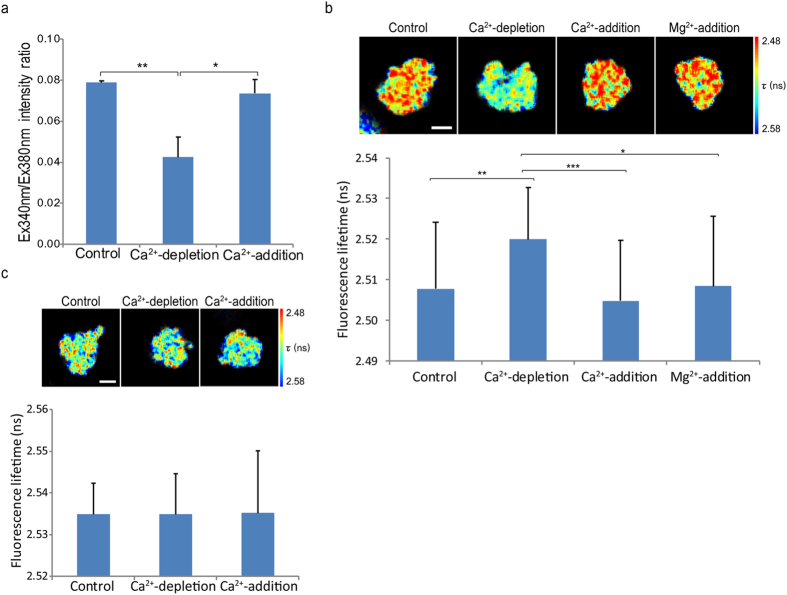
Alteration of chromosome compaction *in vivo* by manipulating intracellular calcium levels. (**a**) Concentration of intracellular Ca^2+^ measured by Fura-2 AM in nocodazole-arrested HeLa^H1.2-GFP^ cells in Ca^2+^-depleted and Ca^2+^-re-added medium. The bar graph shows the intensity ratio of Fura-2 excited at 340 and 380 nm, which represent intracellular calcium levels (*n* = 220 cells). (**b**) Mitotic-arrested HeLa^H2B-2FP^ cells were incubated in Ca^2+^-depleted medium followed by Ca^2+^-added medium. Chromosome compaction was quantified by FLIM-FRET analysis using 2P-FLIM (with 920 nm excitation). Pseudocolours represent fluorescence lifetime (τ) of EGFP. A graph shows mean fluorescence lifetime of EGFP, τ (*n* = 25 cells for control, *n* = 51 cells for Ca^2+^-depletion, *n* = 43 cells for Ca^2+^-addition and *n* = 27 cells for Mg^2+^-addition). (**c**) Mitotic-arrested HeLa^H2B-EGFP^ cells were treated with the same treatments described in (**b**). A graph shows mean fluorescence lifetime from 45 cells in control and Ca^2+^-depletion. Error bars indicate standard deviations. Bar, 5 μm.

To further clarify the role of Ca^2+^ in chromosome compaction in living cells, we examined the compaction of chromosomes exposed to different Ca^2+^ concentrations using HeLa^H2B-2FP^ cells arrested in the prometaphase stage by nocodazole treatment. Nocodazole treatment did not affect chromosome compaction (Supplementary Fig. 3a). Next, we treated nocodazole-treated cells with BAPTA and ionomycin to deplete intracellular Ca^2+^ and CaCl_2_ together with ionomycin, which restored the intracellular Ca^2+^ levels (Fig. 2a). Ca^2+^-depletion significantly increased mean fluorescence lifetime of EGFP from 2.509 ± 0.016 ns to 2.520 ± 0.013 ns, indicating chromosome decondensation. Re-addition of Ca^2+^ caused chromosome recompaction because mean fluorescence lifetime of EGFP was dropped to 2.505 ± 0.015 ns (Fig. 2b). The same treatment was also applied to HeLa^H2B-EGFP^ cells expressing only H2B-EGFP, a donor fluorophore. No significant difference in fluorescence lifetime of EGFP in HeLa^H2B-EGFP^ upon Ca^2+^ concentration (Fig. 2c) indicated that Ca^2+^ was solely responsible for the changing of EGFP fluorescence lifetime in HeLa^H2B-2FP^ cells.

In substitution of Ca^2+^, re-addition of Mg^2+^, after Ca^2+^-depletion also shortened fluorescence lifetime of EGFP (Fig. 2b) indicating that Ca^2+^ contributes in chromosome condensation simply as a cation which possibly directly strengthens the neutralization of negatively charged DNA and promotes chromosome condensation as demonstrated by nucleosome arrays in previous studies^18^,^19^,^20^.

In addition to FLIM-FRET, the dynamics of chromosome condensation in living cells were measured using fluorescence intensity^35^. HeLa^H1.2-EGFP^ cells, which express EGFP more intensely than HeLa^H2B-2FP^ cells, received the same treatment. Three-dimensional chromosome images of HeLa^H1.2-EGFP^ cells were acquired during Ca^2+^ depletion/addition and projected into two-dimensional images. Chromosome compaction was calculated by normalizing the mean fluorescence intensity of H1.2-EGFP with the first time point (*t* = 0 min). The normalized mean intensity of EGFP decreased when Ca^2+^ was reduced and increased again once Ca^2+^ was re-added (Supplementary Fig. 3b). These *in vivo* results confirmed that chromosome compaction/decompaction is influenced by the concentration of Ca^2+^ in living cells.

### Structural contribution of calcium to chromosomes

**Figure 3:**
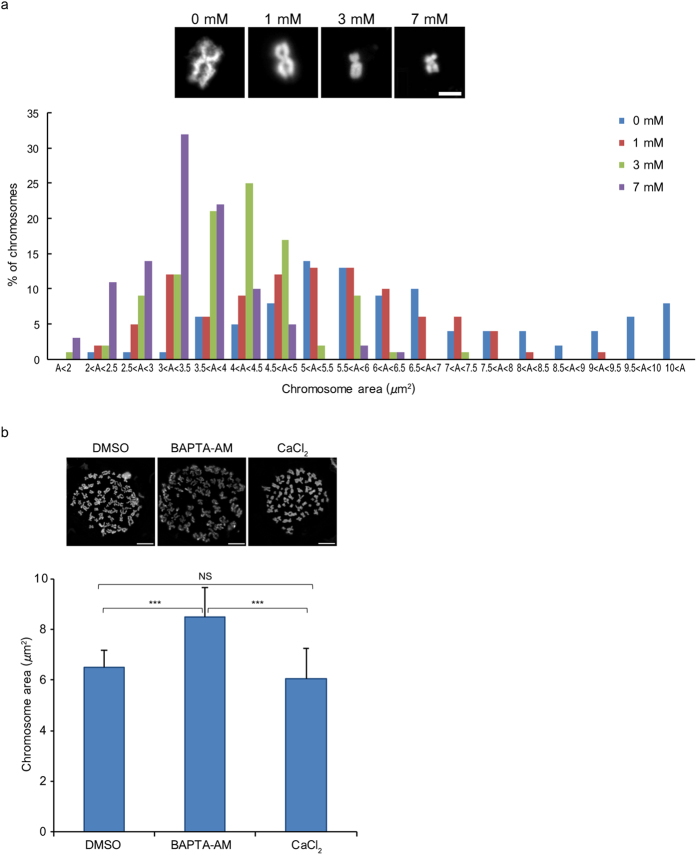
Chromosome expansion induced by calcium. (**a**) Effect of calcium on chromosome area. A histogram represents chromosome area of PA chromosomes treated with 0, 1, 3, and 7 mM CaCl_2_ in XB buffer (*n* = 100 chromosomes). (**b**) Average chromosome area in chromosome spreads after depletion (BAPTA-AM) and re-addition (CaCl_2_) of calcium (*n* = 7 chromosome spreads from three experiments). Error bars indicate standard deviations. Bars: (**a**) 2 μm and (**b**) 10 μm.

The alteration of chromosome compaction by Ca^2+^ was further investigated using isolated chromosomes. Chromosomes isolated from HeLa S3 cells using the polyamine method (PA chromosomes) were treated with various concentrations of CaCl_2_, and morphological changes were observed using optical microscopy. In the absence of CaCl_2_, chromosomes expanded, increasing the chromosome areas. At the higher CaCl_2_ concentrations, chromosomes became compact (Fig. 3a). Chromosomes reached their most compact status at 7 mM CaCl_2_, and further increases in CaCl_2_ concentration did not affect the chromosome area (Supplementary Fig. 4).

A similar phenomenon was observed in chromosome spreads prepared from HeLa S3 cells; the average chromosome area of spreads prepared from BAPTA-AM-treated cells was larger than those prepared from DMSO-treated cells. Interestingly, when these expanded chromosomes were incubated with buffer containing CaCl_2_, the average chromosome area returned to control levels (Fig. 3b). This suggests that Ca^2+^ depletion leads to chromosome expansion and that chromosome compaction can be altered simply by adding Ca^2+^.

### Alteration of chromosome higher-order structure by calcium

**Figure 4:**
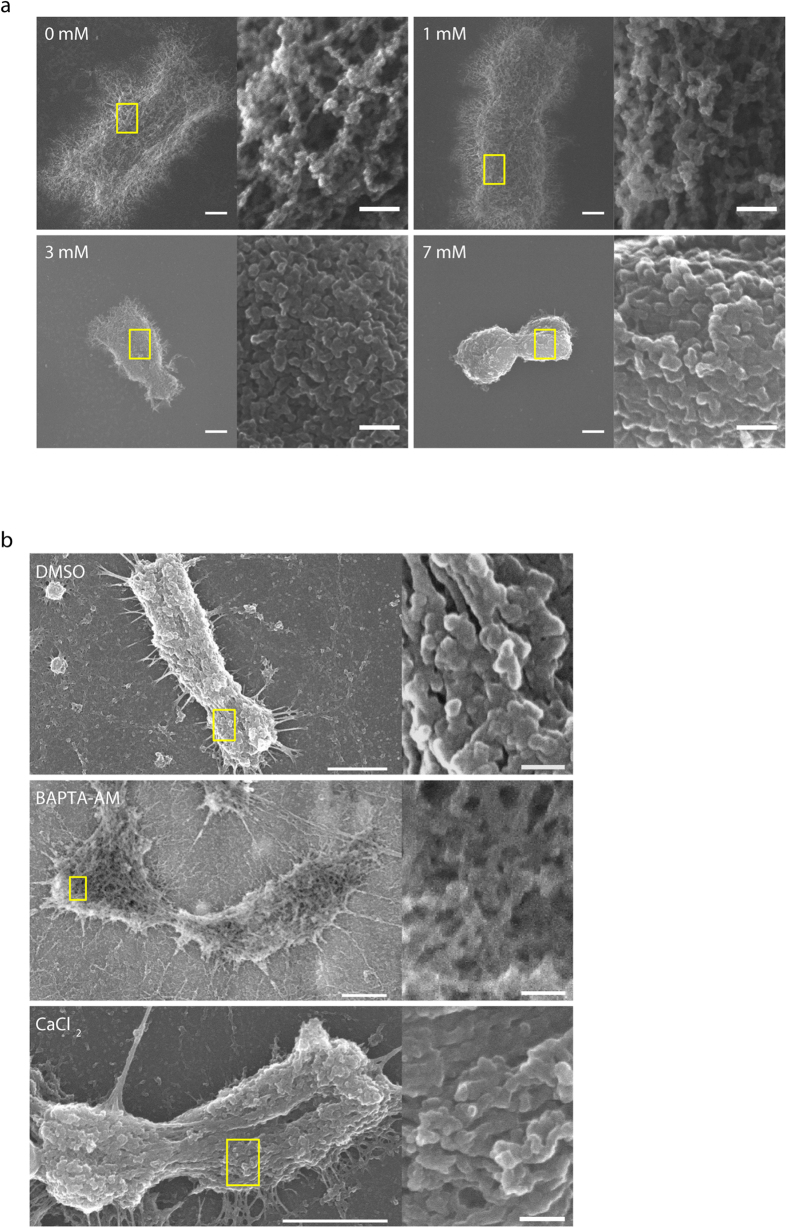
Calcium alters chromosome structure in a concentration-dependent manner. (**a**) SEM images of PA chromosomes treated with 0–7 mM CaCl_2_ in XB buffer. (**b**) SEM images of chromosome structure upon the depletion and re-addition of calcium. Bars: (left) 1 μm and (right) 100 nm.

Using scanning electron microscopy (SEM), chromosome structures were visualized at high resolution. At high CaCl_2_ concentrations (≥3 mM), chromosomes were compact, and chromosome surfaces were smooth. Globular structures were observed on chromosomes. At low CaCl_2_ concentrations (<3 mM), chromosomes expanded and became more fibrous (Fig. 4a). In DMSO-treated cells, chromosomes were compact, and chromosome surfaces contained globular structures. In Ca^2+^-depleted cells, chromosome spreads had fibrous structures on the surface. Chromosome compaction was reversed by incubating Ca^2+^-depleted chromosome spreads in CaCl_2._ This reconstructed the compact globular structure (Fig. 4b). Taken together with our optical microscopic observations, these results show that chromosome compaction/expansion induced by the presence/depletion of Ca^2+^ involves a transition of chromatin between fibrous compact globular structures within chromosomes.

## Discussion

**Figure 5:**
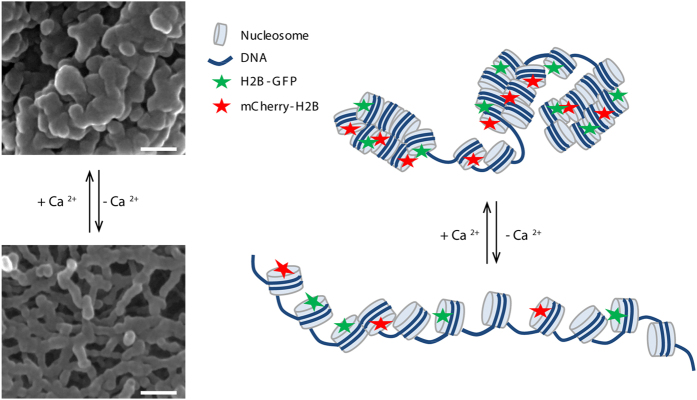
A model for the effect of Ca^2+^ on chromosome condensation. Schematic chromatin structure manipulated by Ca^2+^: interchange of chromatin structure between compact globular structure and fibrous structure could be induced by removal and addition of Ca^2+^, respectively. Bar, 100 nm.

During cell division, chromosomes are duplicated by DNA replication, producing daughter cells with their own complete set of genetic information. Chromosome condensation and segregation during mitosis is regulated by several factors^36^. Here we found that Ca^2+^, a universal secondary messenger in cells, is important for mitotic progression and promoting chromosome condensation. Using high-resolution electron microscopy, we demonstrated that Ca^2+^ is required for mitotic chromosome compaction by influencing the transition of chromatin from a fibrous structure to a compact globular structure. In addition, FLIM-FRET experiments confirmed that Ca^2+^ controls the transition between decondensed and condensed chromatin structures in living cells (Fig. 5).

The packaging of nucleosome fibres into more compact chromatin structures can be demonstrated using reconstituted nucleosomes^18^,^20^ or chromatin isolated from cells^37^. However, these results cannot always be extrapolated to the *in vivo* situation and should be confirmed using suitable methods such as fluorescence intensity measurement and FLIM-FRET analysis. Incubation of prometaphase-arrested cells in Ca^2+^-depleted medium decreased chromosome compaction but did not completely decondense the chromosomes like the *in vitro* situation because other condensation factors in the cytosol would maintain chromosome organization. The fluorescence intensity method and FLIM-FRET analysis demonstrated that chromosome compaction was re-occurred by the re-addition of Ca^2+^. Because FRET requires two fluorophores to be within 10 nm apart, the FLIM-FRET method provides higher spatial resolution that is close to the dimensions of chromatin. In contrast, the fluorescent intensity method shows the density of fluorophores. Therefore, the FLIM-FRET analysis should be able to reflect the change in chromatin structure more sensitively than the ordinary fluorescence intensity measurement.

Ca^2+^ concentrations are critical for the progression of entire cell cycle, especially mitosis. Intracellular Ca^2+^ concentrations increase from mitotic entry in response to ionositol-1,4,5-triphosphate (InsP_3_), which releases Ca^2+^ from intracellular storage organelles^38^. Increasing Ca^2+^ concentrations during mitosis guide the cellular progression through mitosis, by mechanisms including NEB, chromosome condensation, spindle fibre formation and sister chromatid separation^21^,^26^,^39^. Ca^2+^ is a universal intracellular secondary messenger; therefore, the prolonged prometaphase stage caused by Ca^2+^ depletion might be explained by effects other than the compaction of chromosomes. Since Ca^2+^ works as a secondary messenger, several proteins including CaMKII can be activated or inhibited by changes in intracellular Ca^2+^ levels^23^. Ca^2+^ depletion and CaMKII inhibition prevent transition from metaphase to anaphase by preventing APC/C activation. Inactivation of APC/C perturbs the degradation of securin and cyclin B, arresting cells in metaphase^40^,^41^. Here we also showed that Ca^2+^ depletion and CaMKII inhibition delayed progression into prometaphase stages although this may involve two different mechanisms. Ca^2+^ depletion reduced the neutralization of negatively charged DNA, which interfered with chromosome condensation. Inhibition of CaMKII probably increased chromosome compaction by preventing its direct binding to chromatin^42^ or interacting with chromatin remodelling proteins^43^.

A chromosome is a polyelectrolyte complex; therefore, chromosome compaction should depend on how well the negative charges of DNA are neutralized to allow condensation. Sixty percent of negative charges of DNA are neutralized by histone binding while the remaining 40% interacts with other positively charged molecules^19^. The most abundant intracellular cations are Na^+^, K^+^, Mg^2+^, and Ca^2+^. Na^+^ and K^+^ levels are similar in interphase and mitotic nuclei, while Mg^2+^ and Ca^2+^ levels are changeable because they are released from storage organelles to chromatin during mitosis^22^. Ca^2+^ levels in chromosomes increase five times in mitosis compared with interphase^22^. This cell cycle-dependent change in the distribution of Ca^2+^ during mitosis strongly suggests that it contributes to chromosome condensation. Ca^2+^ depletion caused chromosome decompaction, which might prevent or delay the attachment of chromosomes to spindle fibres, resulting in prolonged chromosome alignment at the spindle equator and defective chromosome alignment. Reconstituted nucleosome arrays have demonstrated that compaction is induced by cations including mono/divalent cations, or polyamines such as sperimidine^3+^ and spermine^4+ ^18,20, In contrast to monovalent cations which only caused nonspecific shielding of the negative charges of DNA^44^, the shielding of DNA negative charge by a divalent cation, like Ca^2+^, is more effective because it forms a dense layer of counter ions not only around nucleosomal and linker DNA but also extended to unburied regions of histone tails and exposed chromatin surface, leading to more compact oligonucleosomes^17^. Ca^2+^ might also have specific binding sites in the histone core that confine the mobility of DNA on histones in tailless nucleosomes, stabilizing histone–DNA interactions^45^. Moreover, Ca^2+^ might boost chromosome compaction by inhibiting the catalytic activity of Topo IIα and promoting the structural contribution of Topo IIα as a chromosome scaffold protein^22^.

Even though higher amount of Ca^2+^ was found on metaphase chromosomes, Ca^2+^ and Mg^2+^ were detected throughout chromosome in all stages of mitosis with maximal concentration at metaphase^30^. In polytene chromosomes, both Ca^2+^ and Mg^2+^ colocalized with ^81^Br^−^ bands which indicate AT-rich regions of DNA^30^. When tails of core histones were removed, stable histone-DNA interactions were achieved only by the addition of Ca^2+^ and Mg^2+^, but not monovalent cations or heavy metal ions^44^. All these reports suggest that Ca^2+^ and Mg^2+^ should play similar role in promoting chromosome compaction.

The distinct chromosome condensation dynamics in control and Ca^2+^-depleted cells suggests that Ca^2+^ promotes chromosome compaction after NEB. This is in contrast to other chromosome condensation factors, chromosome scaffold proteins and PMTs that bind to chromatin throughout cell cycle, except for condensin I, which binds to chromosome after NEB^46^,^47^. Chromosome condensation is mostly achieved before condensin I binds to chromosomes; therefore, this protein likely promotes lateral compaction and shortens the chromosome axis^47^. Ca^2+^ and condensin I bind to chromosomes later than other factors; therefore, Ca^2+^ might assist condensin I to promote chromosome compaction.

In conclusion, Ca^2+^ maintains the structural integrity of chromosomes. Ca^2+^ is important for mitotic chromosome condensation after NEB and might promote lateral compaction of chromatin fibres together with condensin I. Lack of Ca^2+^ during mitosis disrupts the organization of mitotic chromosomes, causing defects in the progression of cell cycle through mitosis.

## Materials and Methods

### Cell culture

HeLa S3 cells, HeLa cells expressing EGFP-tagged histone H1.2 (HeLa^H1.2-EGFP^), HeLa cells expressing histone H2B tagged with EGFP at the C-terminus (HeLa^H2B-EGFP^)^48^ and HeLa cells expressing histone H2B tagged with EGFP at the C-terminus and mCherry at the N-terminus (HeLa^H2B-2FP^), were used. HeLa S3 cells were cultured in RMPI 1640 medium supplemented with 10% foetal bovine serum. HeLa^H1.2-EGFP^ and HeLa^H2B-2FP^ were cultured in Dulbecco’s Modified Eagle Medium (DMEM) supplemented with 10% foetal bovine serum.

HeLa^H2B-2FP^ was established according to Llères *et al*.^32^. Briefly, pmCherry-C1-H2B vector was constructed by inserting a H2B sequence into the pmCherry-C1 vector. Then, pmCherry -C1-H2B was transfected into HeLa^H2B-EGFP^ using X-tremeGene HP (Roche) for 48 h. Cells expressing mCherry-H2B were selected by 800 μg/ml G418 (Roche).

### Live cell imaging and chromosome condensation analysis by analysing fluorescence intensity

HeLa^H2B-2FP^, HeLa^H2B-EGFP^ and HeLa^H1.2-EGFP^ cells cultured in 35-mm poly-l-lysine-coated glass bottom dishes were synchronized with double thymidine blocking. Cells were arrested at either prometaphase using nocodazole (Sigma, 80 ng/ml) or metaphase using MG132 (Calbiochem, 20 μM). Before performing time-lapse observation, the medium was discarded and replaced with phenol red-free DMEM containing 10% foetal bovine serum, 4 mM l-glutamine, and 10 mM HEPES for microscopic observation. Cells were monitored using a DeltaVision deconvolution microscope equipped with a CO_2_ chamber at 37 °C (IX71, Olympus, Tokyo, Japan). The system was fitted with a 60 × 1.35 UApo/340 Iris oil immersion objective lens (Olympus). Ca^2+^ was depleted by adding (acetoxymethyl)-l,2-bis(o-aminophenoxy) ethane N,N,N′,N,′-tetra-acetic acid, BAPTA-AM (Dojindo, 25 μM), a membrane permeable Ca^2+^ chelator, to the medium. CaMKII was inhibited by adding KN93 (Calbiochem, 10 μM) to the medium. To manipulate intracellular Ca^2+^, cells were incubated in culture medium containing either 10 mM BAPTA, 5 mM CaCl_2_ or 5 mM MgCl_2_ in the presence of the Ca^2+^ ionophore ionomycin, for 1.5 h to decrease and increase intracellular Ca^2+^ levels, respectively.

Time-lapse three-dimensional images of whole HeLa^H1.2-GFP^ chromosomes were collected with 2-μm spacing between stacks. Raw 3D images were deconvoluted using constrained iterative deconvolution and converted to 2D images with maximum projection using softWoRx (AppliedPrecision). All images were processed using ImageJ (National Institute of Health). The quantification of chromatin compaction by measuring the fluorescence intensity of EGFP-tagged histone H1.2, which binds to chromatin specifically, was previously described^35^. Chromosome areas were defined by scaling the grey value to a specific value that distinguished the whole chromosome areas from the background. Total fluorescence intensity and total number of pixels in the chromosome area were measured. Mean fluorescence intensities in the chromosome area of each image were calculated by dividing total fluorescence intensity with total number of pixels in chromosome area, and normalized with that of the first image to generate the normalized mean fluorescence intensity, representing degrees of chromatin compaction.

### Measurement of intracellular Ca^2+^ levels

Calcium concentration was measured using the membrane permeable Ca^2+^-specific ratiometric dye, Fura-2 AM. HeLa^H1.2 EGFP^ cells were cultured in a glass bottom dish and treated with nocodazole before treating with 1 μM Fura-2 AM (Dojindo) for 45 min at room temperature and incubating in nocodazole for a further 20 min to allow complete hydrolysis of AM esters inside the cells. Immediately after replacing with new phenol red-free medium, the cells were placed on the temperature-controlled stage of the inverted microscope equipped with a CO_2_-chamber at 37 °C (Olympus IX71) and a 40 × 1.35 UApo/340 Iris oil immersion objective lens. Fluorescence images were acquired over areas containing 10–15 mitotic cells by exciting internalized Fura-2 sequentially at 340 and 380 nm and measuring emission at 510 nm. Quantification of fluorescence at 510 nm after excitation at 340 and 380 nm (F340/F380 ratio) was used as an index for intracellular Ca^2+^ levels using ImageJ. Regions of interest (ROIs) were drawn around chromosome regions.

### Fluorescence lifetime measurement using TCSPC

Quantification of chromatin compaction in living cells was done using two different systems of two-photon fluorescence lifetime imaging microscopy (2P-FLIM). Measuring of chromatin compaction of the first system was previously described^31^,^32^. FLIM was performed using a confocal laser scanning microscope system (TCS SP5, Leica, Germany) equipped with a TCSPC module SPC-830 (Becker & Hickl GmbH). EGFP was excited with an 880 nm MP laser (Mai Tai, Spectra-Physics, CA, USA) and captured through 63× HCX PL APO ibd.BL objective lens (Leica) with 491–550 nm bandpass in a 256 × 256 pixel format for fluorescence lifetime imaging (90-s scanning time for each image). Laser power, sensitivity of the detector, and pinhole size were adjusted to give a photon count rate between 5 × 10^4^ − 1 × 10^5^ photons/s.

For the second 2P-FLIM system, measuring of fluorescence lifetime was described previously in detailed^49^. EGFP was excited with 920 nm Ti-sapphire laser (Mai Tai) and acquired through 60 × 0.9 NA objective lens (Olympus) with a 256 × 256 pixel format.

The obtained fluorescence decay curve in each pixel was fitted with single (for HeLa^H2B-EGFP^) or double (for HeLa^H2B-2FP^) exponential function using SPCImage software (Becker & Hickl) and custom MATLAB software for the first (880 nm excitation) and the second (920 nm excitation) 2P-FLIM system, respectively. The mean fluorescence lifetime in the ROI was calculated to generate a fluorescence lifetime map. The variation in the fluorescence lifetime is represented by pseudocolours.

### Preparation of metaphase chromosome spread

HeLa S3 cells were grown until 80% confluency. Cells were arrested in metaphase with 0.1 μg/ml colcemid for 3 h. To deplete intracellular calcium, cells were treated with 25 μM BAPTA-AM for 2 h. Cells were collected for metaphase-chromosome spread as follows: cells were treated with hypotonic solution (75 mM KCl) for 15 min at 37 °C and were cytospun onto coverslips. Then cells were permeabilized by 0.5% Triton X-100 in PBS for 15 min. Some cells were incubated with 5 mM CaCl_2_ for 30 min before observation using either a fluorescence microscope or SEM.

### Fluorescence microscopy

Chromosome spreads and isolated PA chromosomes were stained with DAPI and observed using fluorescence microscopy with a 100 × 1.40 Plan-APOCHROMAT Iris oil immersion objective lens (Zeiss). The average chromosome areas were measured using ImageJ software. Lines were drawn around chromosomes. In chromosome spreads, the total pixels in the chromosome area were counted and divided by the number of chromosomes for calculating an average chromosome area.

### Scanning electron microscopy

Chromosomes were fixed with 2.5% (v/v) glutaraldehyde at 4 °C overnight, and 1% tannic acid for 10 min at room temperature, respectively. Then chromosomes were postfixed with 2% OsO_4_ for 15 min and washed using Milli-Q water. Sample dehydration was performed using an ethanol series (70%, 100%, and 100%) and subjected to critical point drying using 3-methylbutyl acetate, followed by osmium coating and observation by high vacuum SEM (HITACHI, S-5200) with a secondary electron voltage of 10 kV.

### Statistical analysis

Statistical analysis was carried out in R using either the student *t*-test or the Mann-Whitney-Wilcoxon test to compare the mean values. For the student *t*-test, the test of normality was done using the Shapiro-Wilk test. ‘*’, ‘**’, and ‘***’ indicated *p* < 0.05, 0.01, and 0.001, respectively.

## Additional Information

**How to cite this article**: Phengchat, R. *et al*. Calcium ions function as a booster of chromosome condensation. *Sci. Rep.* **6**, 38281; doi: 10.1038/srep38281 (2016).

**Publisher's note:** Springer Nature remains neutral with regard to jurisdictional claims in published maps and institutional affiliations.

## Supplementary Material

Supplementary Information
