# Supplementary material for: Calcium ions function as a booster of chromosome condensation
Source: Sci Rep. 2016 Dec 2;6:38281. doi: 10.1038/srep38281 (PMC5133622; doi:10.1038/srep38281)
Supplement: Supplementary Information [file srep38281-s1.pdf]

Calcium ions function as a booster of chromosome condensation

Rinyaporn Phengchat, Hideaki Takata, Kenichi Morii, Noriko Inada, Hideji Murakoshi, Susumu Uchiyama, Kiichi Fukui

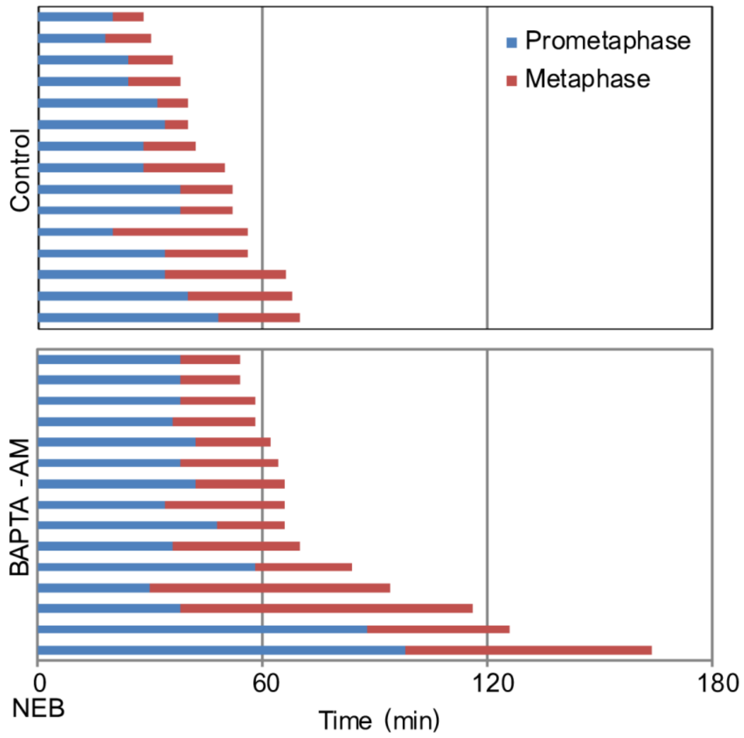

Supplementary Figure 1. Mitotic progression in 15 control and Ca^{2+}-depleted cells selected at random.

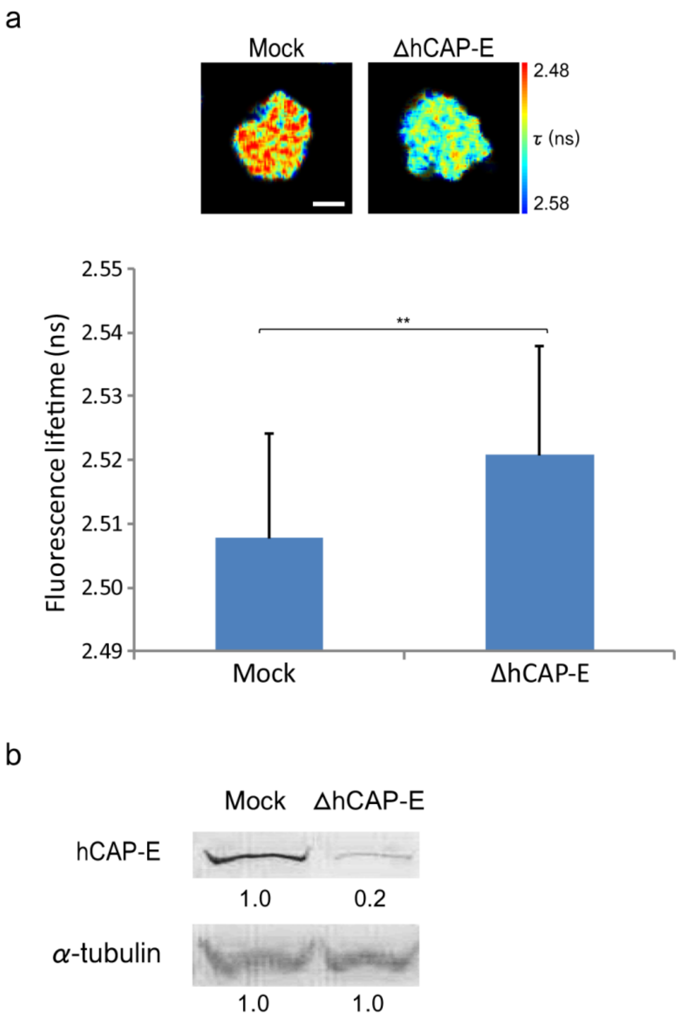

Supplementary Figure 2. (a) Compaction of chromosomes mitotic-arrested condensin-depleted (ΔhCAP-E) cells was quantified by FLIM-FRET analysis using 2P-FLIM with 920 nm excitation. Pseudocolours in the images represent fluorescence lifetime of EGFP. A column graph showed mean fluorescence lifetime from 25 cells in mock, 61 cells ΔhCAP-E. (b) Western blot of hCAP-E protein from whole cell extract of HeLa^{WT} after the depletion of hCAP-E by siRNA transfection. Alpha-tubulin was used as control. The relative band intensity ratios to mock are shown below. Error bars indicate standard deviations. Bar, 5 µm.

a

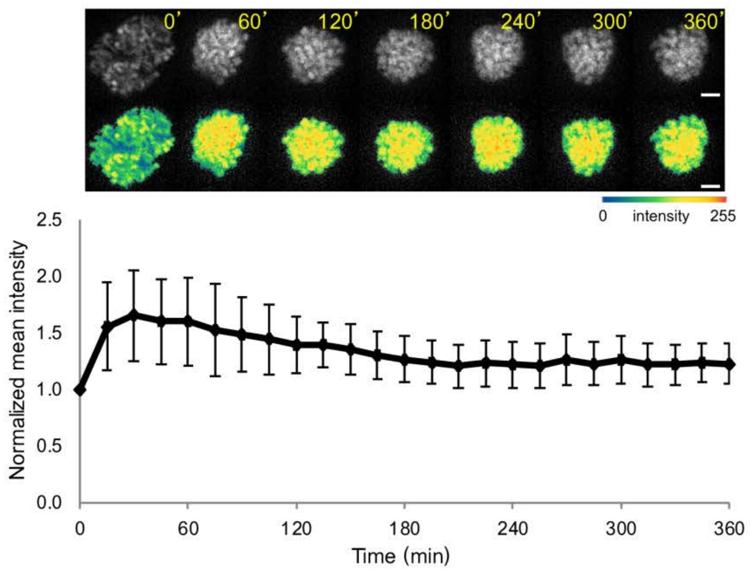

b

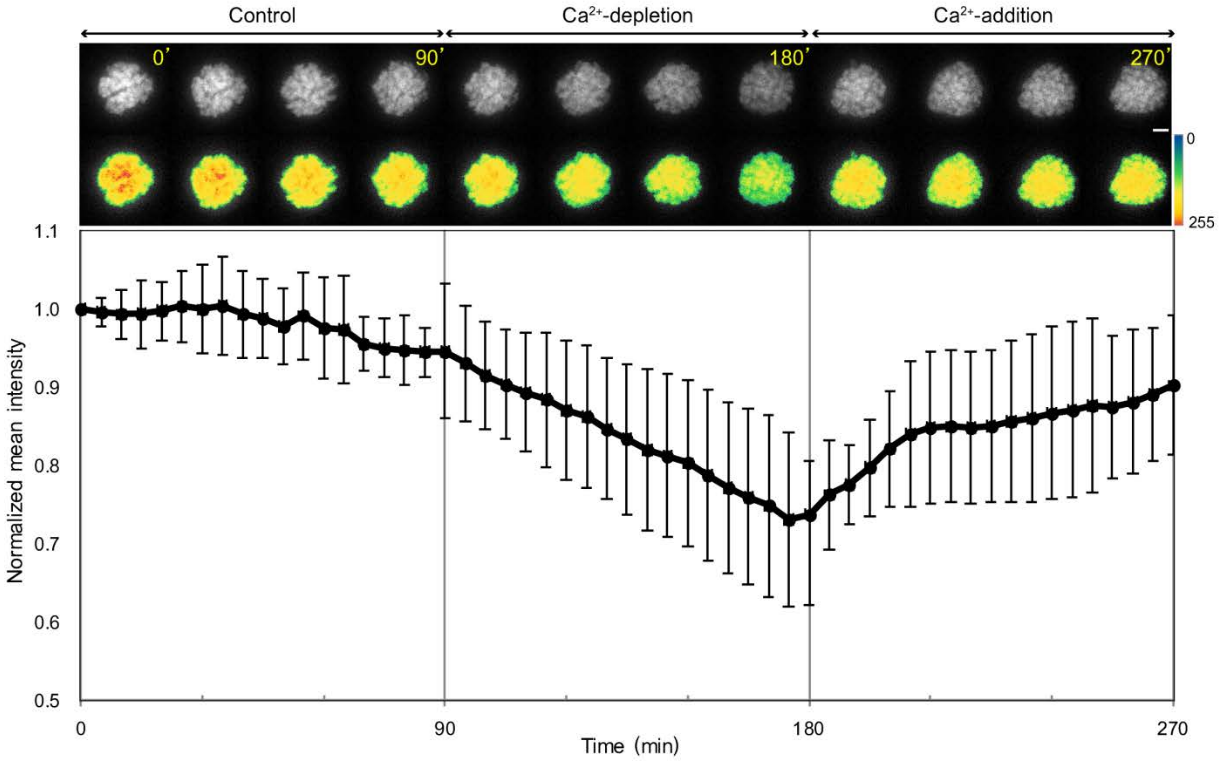

Supplement Figure 3. Intracellular Ca^{2+}-altered chromatin compaction levels *in vivo*. (**a**) Chromatin compaction levels of nocodazole-arrested $HeLa^{H1.2\text{-}GFP}$ cells were measured using fluorescent intensity of EGFP. Three-dimensional images of whole chromosomes starting from NEB were acquired every 15 min for 6 h. Plot graph

showing the quantification of chromosome condensation under mitotic arrest (*n* = 6 cells). (**b**) Fluorescent images of mitotic arrested $HeLa^{H1.2\text{-}GFP}$ sequentially incubated in Ca^{2+}-depleted and Ca^{2+}-re-added medium. A graph represents the quantification of chromatin compaction levels as normalized mean fluorescent intensity of EGFP (*n* = 15 cells). Error bars indicate standard deviations. Bar, 5 µm.

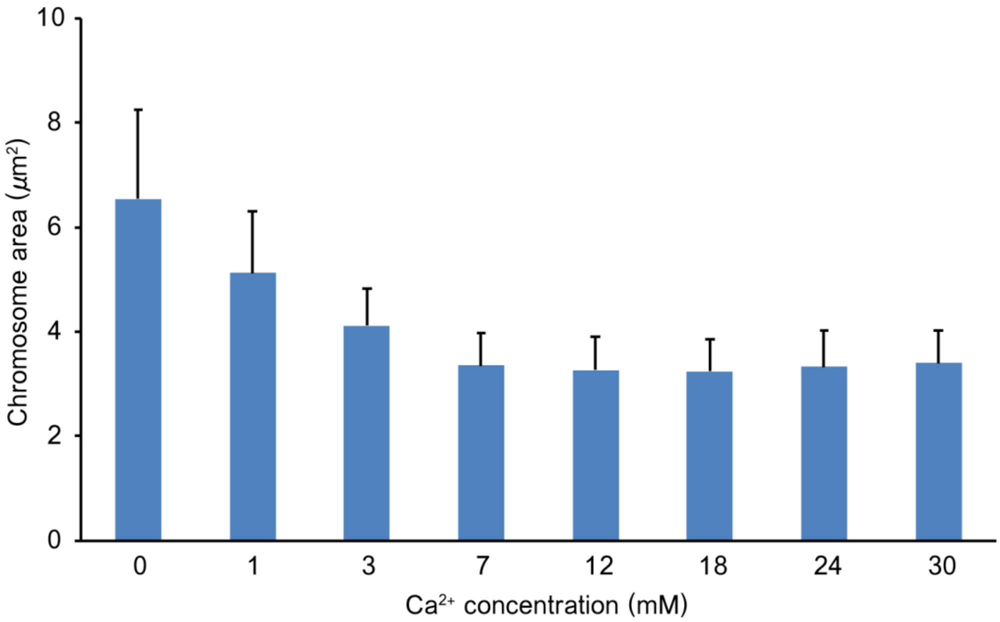

Supplementary Figure 4. Average area of PA chromosomes treated with XBE buffer containing various concentrations of $CaCl_2$ (n = 100 chromosomes).
